# Superbug Hideout: Finding MRSA in U.S.Wastewater Treatment Plants

**DOI:** 10.1289/ehp.120-a437a

**Published:** 2012-11-01

**Authors:** Rebecca Kessler

**Affiliations:** Rebecca Kessler is a science and environmental journalist based in Providence, RI.

For many years, the “superbug” methicillin-resistant *Staphylococcus aureus* (MRSA) was confined to hospital patients, but since the 1990s it has been infecting otherwise healthy people in settings such as schools and locker rooms. Researchers now report the discovery of MRSA in U.S. wastewater treatment plants (WWTPs), identifying another possible environmental reservoir for the bacteria [*EHP* 120(11):1551–1558; Rosenberg Goldstein et al.]. People shed MRSA from their nostrils and skin and in their feces, which makes wastewater a likely vector for the bacteria. Previous research had identified MRSA in Swedish WWTPs, but no studies had been conducted on U.S. facilities.

The researchers collected wastewater samples throughout the treatment process at two mid-Atlantic and two Midwestern WWTPs. MRSA, as well as pathogenic methicillin-susceptible *Staphylococcus aureus* (MSSA), were present at all four WWTPs, with MRSA detected in 50% of all samples and MSSA detected in 55%. Genetic testing of the MRSA isolates revealed traits common among exceptionally virulent community-acquired strains circulating in the United States. Most MRSA isolates and 29% of MSSA isolates showed resistance to two or more classes of antibiotics, including several that the U.S. Food and Drug Administration has specifically approved for treating MRSA infections.

MRSA and MSSA each were detected in 83% of untreated influent samples. But the percentage of MRSA- and MSSA-positive samples decreased as water treatment progressed, making up only 8% and 17% of samples of fully treated effluent, respectively. There was some evidence that MRSA isolates that survived initial stages of treatment may have been resistant to more antibiotics and may have had a greater prevalence of a gene associated with virulence than other isolates in influent samples. However, chlorination, the tertiary stage in wastewater treatment, appeared to eliminate all MRSA. Only one WWTP had the bacteria in a subset of final effluent samples, and this occurred only when tertiary chlorination was not carried out.

**Figure:**
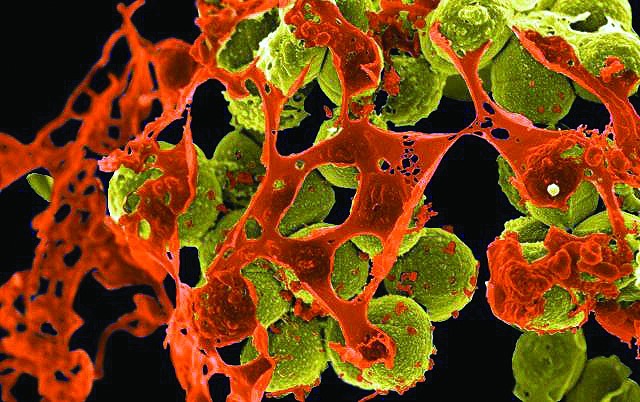
Scanning electron micrograph of MRSA (yellow) surrounded by cellular debris (orange) © National Institute of Allergy and Infectious Diseases

The researchers genetically profiled only a subset of the MRSA isolates, so they may have underestimated the diversity present in the WWTPs. They also did not determine the levels of MRSA and MSSA in the samples. Nevertheless, the study suggests that WWTP workers and people who live, work, or play near the increasing number of agricultural and recreational sites irrigated with reclaimed wastewater could be exposed to MRSA and MSSA. Risk might be reduced through frequent hand washing and the use of gloves among WWTP workers and through tertiary treatment of wastewater that is to be used for irrigation.

